# Spatial epidemiology and serologic cohorts increase the early detection of leprosy

**DOI:** 10.1186/s12879-015-1254-8

**Published:** 2015-11-16

**Authors:** Josafá Gonçalves Barreto, Donal Bisanzio, Marco Andrey Cipriani Frade, Tania Mara Pires Moraes, Angélica Rita Gobbo, Layana de Souza Guimarães, Moisés Batista da Silva, Gonzalo M. Vazquez-Prokopec, John Stewart Spencer, Uriel Kitron, Claudio Guedes Salgado

**Affiliations:** Laboratório de Dermato-Imunologia UEPA/UFPA/Marcello Candia, Av. João Paulo II, 113. Bairro Dom Aristides, Marituba, CEP: 67200-000, Pará Brazil; Universidade Federal do Pará, Campus Castanhal, Marituba, Pará Brazil; Department of Environmental Sciences, Emory University, Atlanta, GA USA; Divison of Dermatology of Internal Medicine Department of Ribeirão Preto Medical School, University of São Paulo, Ribeirão Preto, São Paulo Brazil; Unidade de Referência Especializada em Dermatologia Sanitária Dr. Marcello Candia, Marituba, Pará Brazil; Department of Microbiology, Immunology and Pathology, Mycobacteria Research Laboratories, Colorado State University, Fort Collins, CO USA; Instituto de Ciências Biológicas, Universidade Federal do Pará, Belém, Pará Brazil

**Keywords:** Leprosy, Serology, PGL-I, Spatial epidemiology, Geographic information systems, School children

## Abstract

**Background:**

Leprosy remains an important public health problem in some specific high-burden pockets areas, including the Brazilian Amazon region, where it is hyperendemic among children.

**Methods:**

We selected two elementary public schools located in areas most at risk (cluster of leprosy or hyperendemic census tract) to clinically evaluate their students. We also followed anti-PGL-I seropositive and seronegative individuals and households for 2 years to compare the incidence of leprosy in both groups.

**Results:**

Leprosy was detected in 11 (8.2 %) of 134 school children in high risk areas. The difference in the prevalence was statistically significant (*p* < .05) compared to our previous findings in randomly selected schools (63/1592; 3.9 %). The 2-year follow-up results showed that 22.3 and 9.4 % of seropositive and seronegative individuals, respectively, developed leprosy (*p* = .027). The odds of developing overt disease in seropositive people were 2.7 times that of negative people (*p* < .01), indicating that a follow-up of 10 seropositives has a >90 % probability to detect at least one new case in 2 years. The odds of clinical leprosy were also higher in “positive houses” compared to “negative houses” (*p* < .05), indicating that a follow-up of ten people living in households with at least one seropositive dweller have a 85 % probability to detect at least one new case in 2 years.

**Conclusions:**

Targeted screening involving school-based surveillance planned using results obtained by spatial analysis and targeted household and individual continuous surveillance based on serologic data should be applied to increase the early detection of new leprosy cases.

## Background

Although the World Health Organization (WHO) elimination target has been achieved in 2000, with a global prevalence rate of <1 case/10,000 people, leprosy remains an important public health problem in some specific high-burden pockets [1]. In the most recent global statistics, 206,107 (96 %) of new leprosy cases were reported from only 14 countries, and among them, India, Brazil and Indonesia account for more than 80 % of all new cases [2].

Brazil has one of the highest annual case detection rates in the world (15.4/100,000 people), with 31,044 new cases reported in 2013 [2]. Despite the recent Brazilian economic growth, large pockets of poverty remain, especially in the North, Central-West and Northeast of the country, where leprosy is hyperendemic and underdiagnosed [3, 4]. Approximately half of the Brazilian cases were detected in high-burden municipalities that encompass only 17 % of the total national population [5].

The problem is historic in the state of Pará, in the Brazilian Amazon region, north of the country, where approximately 80,000 new cases were reported during the last 20 years. In 2012, the annual case detection rate of this state reached 50/100,000 people, which was three times the national average (17/100,000) according to official numbers from the Brazilian Ministry of Health. Because of the long incubation period averaging 3–7 years to develop clinical symptoms and the continued spread of infection from asymptomatic individuals, the chain of transmission in these areas continues uninterrupted, with leprosy remaining hyperendemic among children less than 15 years old (6.4 % of new cases, or 1996 of 31,044 reported by WHO for Brazil in 2013 [2]), indicating active foci of infection in the community [3, 6, 7]. Additionally, about 50 % of the population is not covered by the family health strategy, which is responsible for detecting and treating leprosy cases (http://dab.saude.gov.br/portaldab/historico_cobertura_sf.php). This fact may explain the high number of undiagnosed leprosy cases recently discovered in Pará [3, 6]. Based on our survey results in eight different municipalities throughout this state [3], we estimate that there are approximately 80,000 cases among the 2,000,000 public school students who are waiting to be diagnosed in Pará, many of them in difficult to reach areas poorly served by health professionals.

There is no laboratory test that detects all forms of leprosy, but some biomarkers of infection, disease progression and treatment efficacy have been developed since the isolation and characterization in the 1980s of phenolic glycolipid-I (PGL-I), a species-specific antigen from the *M. leprae* cell wall [8, 9]. Serology could potentially be used to detect antibodies against PGL-I to help classify patients for treatment purposes, monitor treatment efficacy, identify the risk of relapse and identify the healthy household contacts (HHC) of leprosy patients who are most at risk of contracting the disease [10].

Anti-PGL-I seropositivity is also a marker of subclinical infection in healthy subjects [11, 12]. A positive test for anti-PGL-I IgM is associated with an 8.6-fold higher risk of leprosy in HHC and a 4.4-fold higher risk in non-contacts [13]. In our recent school-based surveys performed in the state of Pará, we recorded a seroprevalence of 48.8 % among students ranging from 6 to 20 years old, and 4 % of those surveyed were diagnosed based on well-defined clinical signs and symptoms, including loss of sensitivity in hypochromic skin lesions, nerve swelling or pain, and weakness, sensory loss or loss of function associated with nerve damage in the extremities such as the hands and feet [3]. Additionally, it is believed that a healthy carrier, those with *M. leprae* in their noses, might be actively involved in transmission through the shedding of bacilli facilitating its spreading in endemic regions [14].

The recent huge growth in spatial epidemiology is facilitated by improved accessibility of computer-based geographic information systems (GIS) and personal computing improvements in processing speed and user-friendly applications. The combination of these factors has allowed spatial analysis to reach a large number of researchers and health policy-makers [15]. GIS technology and spatial analysis have been applied to identify the distribution of leprosy at the national, regional and local levels [4, 16–18]. Indeed, the WHO recently advocated using GIS as part of the “Final Push” strategy as a management tool to strengthen capacities in surveillance and monitoring of new cases and to monitor epidemiological indicators over time, aiming to identify risk factors and clusters of substantial endemics and to indicate precisely where additional resources should be targeted (http://www.who.int/lep/monitor/gis/en/index.html).

According to the WHO, the introduction of innovative case-finding methods in hard-to-reach areas and population groups, coupled with improved data management, will result in a large increase in detection of new leprosy cases [19]. 1) In our recent studies, we detected a very high rate of previously undiagnosed leprosy and subclinical infection in the state of Pará [3, 6]; and 2) leprosy cases were spatially clustered in hyperendemic pockets, even at a fine intra-town spatial scale [20]. Therefore, the main objective of this study is to describe and evaluate a targeted screening strategy for the early diagnosis of leprosy cases, involving school-based active clinical surveillance in high risk areas determined by spatial epidemiology, accompanied by regular follow-up of targeted HHC and families guided by anti-PGL-I IgM serologic data.

## Methods

### Ethics, consent and permissions

This study adhered to the Declaration of Helsinki and was approved by the Institute of Health Sciences Research Ethics Committee at the Federal University of Pará (protocol number 197/07 CEP-ICS/UFPA). All data were anonymized. A written informed consent to publish was obtained from every individual who accepted to participate in this study.

### Study area

Our study was performed in two municipalities of the state of Pará: Castanhal (1.29° S; 47.92° W) and Oriximiná (1.76° S; 55.86° W); the first is hyperendemic (annual case detection rate ≥40/100,000 people), and the second is highly endemic (annual case detection rate of 20 to 39.99/100,000 people) for leprosy. Castanhal is located 68 km NE of Belém, the capital of Pará, and it is easily accessed via a paved road. In contrast, Oriximiná is located 820 km west of the capital, and it is accessible only by plane or 3 days of travel by boat on the Amazon and Trombetas Rivers. Table 1 presents some relevant demographic and epidemiologic characteristics of each municipality involved in this study.

**Table 1:** Epidemiologic and demographic characteristics of the study area

Municipality	Population (2010)^a^	Number of new cases detected (2006 to 2010)^b^	Annual new case detection rate per 100,000 people (2006 to 2010)^b^	Children among new cases of leprosy (2006 to 2010)^b^	Seroprevalence among students^c^	New cases detected among students^d^
Castanhal	173,149	380	44.4	35 (9.2 %)	66.5 %	4.8 %
Oriximiná	62,794	68	22.3	5 (7.3 %)	42.2 %	4.4 %

^a^Source: Brazilian Institute of Geography and Statistics (IBGE)

^b^Based on the Brazilian Ministry of Health online database—SINAN

^c^Seroprevalence of anti-PGL-I IgM detected in our previous cross-sectional study conducted in 2010 [3]

^d^New cases detected based on clinical examination in our previous cross-sectional study [3]

### Sampling design and methods

Based on our previous clinical and serologic cross-sectional studies conducted in Castanhal and Oriximiná in 2010 (T1) in which we had evaluated 427 HHC and 323 school children (SC) [3, 6], we sampled and re-examined those clinically healthy subjects who tested positive or negative to anti-PGL-I 2 years after (T2) the first evaluation. To be enrolled in the follow-up study, the subject had to be registered as living in the same urban area as at the beginning of the study. In addition to those people evaluated in T1, we included other HHC (people that share the same house or neighbors with frequent presence at that house) that were found in the households at the time of our second visit, although they had not been examined in T1. The sample size was determined by the number of people who we could survey in 1 week of field work during a single visit to each municipality.

The subjects were clinically assessed by an experienced leprologist. Leprosy cases were diagnosed in the field based on clinical signs, including loss of sensation associated with obvious skin lesions detectable by assessment with standard graded Semmes-Weinstein monofilaments test [21]. For operational reasons, slit skin smears were not performed. The cases were classified as indeterminate leprosy, as defined by the Madrid classification [22], if there was only a hypopigmented macule but no detection of nerve involvement, or as one of the five clinical forms defined by the Ridley and Jopling classification system (tuberculoid-tuberculoid (TT), borderline tuberculoid (BT), borderline-borderline (BB), borderline-lepromatous (BL) or lepromatous-lepromatous (LL)) [23]. Cases of indeterminate and TT leprosy were classified as paucibacillary (PB) cases, whereas the other forms were classified as multibacillary (MB) cases. Primary neural leprosy was diagnosed if nerve enlargement associated with functional or sensory loss was detected but no skin signs were present. If only one nerve was affected, the case was classified as PB; two or more enlarged nerves defined the case as MB. The disability grading (DG) was ranked from 0 to 2 (0 = no disability; 1 = loss of sensation in the hand or foot; 2 = visible damage or disability) as determined by clinical examination of the sensory-motor functions using a WHO standardized neurological evaluation [24].

The subjects’ antibody titres of anti-PGL-I IgM were determined by ELISA as described previously using native PGL-I as the antigen [6]. The ELISA cut-off for the test to be considered seropositive was established as an optical density (OD) of 0.295, based on the average plus 3× the standard deviation of the test results from 14 clinically healthy people from the Amazon region, which would be considered an endemic population. The subjects were also interviewed to identify their demographic and socio-economic characteristics. Detailed information about sampling and eligibility criteria for the first examination can be found in Barreto et al. [6].

Additionally, based on the spatial distribution pattern of leprosy cases described in our previous study in Castanhal [20], we selected two elementary public schools located in high risk areas, one located within a cluster of leprosy cases and the other in a hyperendemic census tract, to survey additional SC. We sent invitation letters to the parents of students of three or four classes selected by the director of each of the two schools (approximately 100 students) to attend a meeting with us in which they received general information about aspects of leprosy and an explanation about our project and experimental procedures. We clinically evaluated and collected peripheral blood samples from those students who received permission with a signed consent by a responsible adult family member. When a new case was detected in a student, we scheduled a visit to their house to evaluate their HHC.

### Data management and analysis

The spatial distribution pattern of leprosy cases in Castanhal was determined by combining information from the National Notifiable Diseases Information System (SINAN—http://dtr2004.saude.gov.br/sinanweb), the Brazilian Institute of Geography and Statistics (IBGE—http://www.ibge.gov.br), and by mapping in the field. The residences of people affected by leprosy in the urban area, reported during the last 6 years before our study, were georeferenced with a handheld GPS device (Garmin *e*Trex H, Olathe, KS, USA) to produce detailed maps of the leprosy distribution. Using GIS (ArcGIS 10 - ESRI, Redlands, CA, USA), we drew point pattern maps, calculated the number of cases and the annual case detection rate per urban census tract and identified hyperendemic areas. Additionally, using the software Clusterseer 2.3 (Biomedware, Ann Arbor, MI, USA), we applied Kulldorff’s spatial scan statistics [25] to identify clusters of leprosy (see Barreto et al. for details [20]). All examined SC also had their residential addresses georeferenced with the GPS device to analyse their spatial correlation with reported leprosy cases.

We used Fisher’s exact test to compare the proportion of new cases detected among seropositive and seronegative people or households and Mann–Whitney *U* test to compare the titres of anti-PGL-I IgM among the groups. We also calculated the odds ratio to analyse the probability of developing disease and the number needed to harm (NNH) based on the seropositivity.

## Results

### Follow-up of individuals

Of the 750 people initially evaluated in T1, we were able to re-examine 254 (33.8 %) 2 years later (T2). Our sample included 94 males and 160 females and 143 HHC and 111 SC. The age of participants ranged from 5 to 80 years (mean = 20, SD = 14.1), with 44 % of individuals in the age group <15 years. The main reasons for non-participation in the follow-up were: (1) families that had moved to unknown addresses inside the same city (2), families that moved to other cities or states and (3) subjects that were not at home at the time of our visit.

**Fig. 1:**
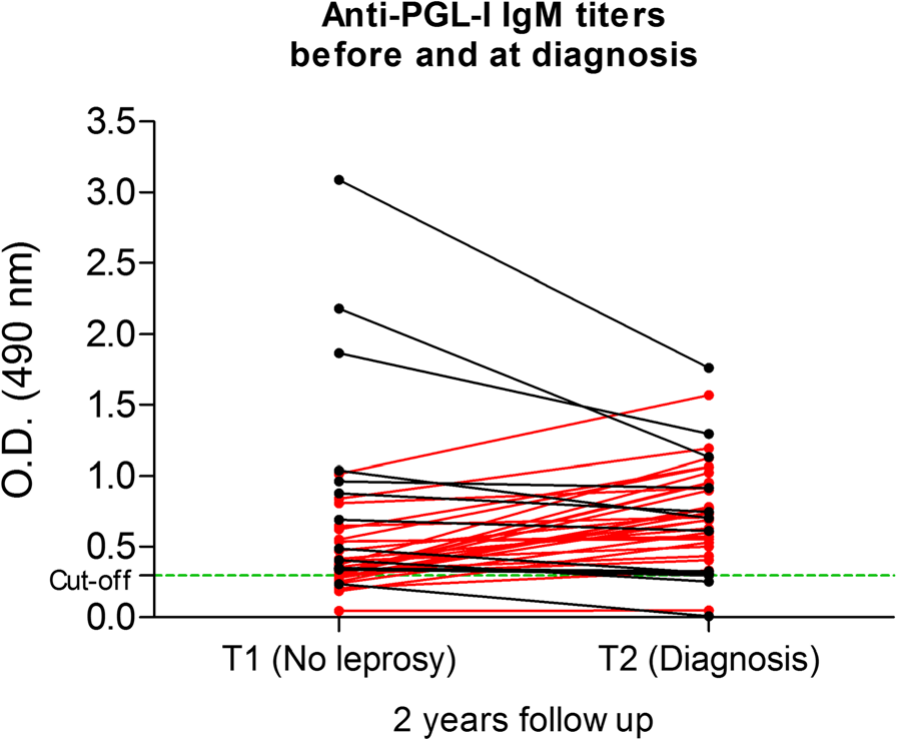
Anti-PGL-I titres before and at diagnosis for people detected with leprosy at 2 years follow-up. The red lines/dots represent those people who showed an increase in their IgM titres (significant increase, *p* < 0.001), whereas black lines/dots indicate those who showed a decrease in their titres (not significant decrease, *p* > 0.2)

In T2, 43 people of 254 (16.9 %) were clinically diagnosed with leprosy. The incidence was significantly higher (*p < 0.05 -* Fisher’s exact test) among those who tested positive to anti-PGL-I in T1 (Table 2). The odds of developing overt leprosy in seropositive people were 2.7 times higher than for seronegative individuals (95%CI = 1.29–5.87; *p* < 0.01), indicating that a follow-up of 10 seropositives has a >90 % probability to detect at least one new case in 2 years. Figure 1 shows the progression of the antibody titration from T1 (no leprosy) to T2 (diagnosis). Of those 43 new cases, 29 (67.4 %) showed a significant increase in their IgM titres (mean increase = 110 %, SD = 80 %; median OD value in T1 = 0.333, IQR = 0.251; median value in T2 = 0.686, IQR = 0.353; *p <* 0.001 by the Mann–Whitney *U* test). The decrease observed in the other 14 subjects was not significant (mean decrease = 30 %, SD = 20 %; median OD value in T1 = 0.956, IQR = 1.755; median value in T2 = 0.723, IQR = 0.947; *p* > 0.2 by the Mann–Whitney *U* test). Considering all 43 new cases, there was a significant increase (*p <* 0.01 by the Mann–Whitney *U* test) in the anti-PGL-I IgM titres from T1 (median OD value = 0.371, IQR = 0.344) to T2 (median OD value = 0.702, IQR = 0.542). During the first evaluation, 33 of the 43 (76.7 %) tested positive to anti-PGL-I, whereas at diagnosis, 39 (90.7 %) were seropositive.

**Table 2:** Follow-up results of individuals evaluated twice (T1 and T2) in the cohort

Serology (T1)^a^	Households visited	People examined	New cases detected in T2 (%)^b^	Paucibacillary	Multibacillary
Positive	113	148	33 (22.3 %)	7	26
Negative	76	106	10 (9.4 %)	2	8
Total	131^c^	254	43 (16.9 %)	9	34

^a^T1 = First evaluation. T2 = second evaluation performed 2 years later

^b^The difference is statistically significant (*p* = 0.027). Fisher’s exact test

^c^At most times, both positive and negative subjects shared the same household

**Table 3:** Follow-up results of subjects evaluated in “positive and negative houses” at T1 and T2^**a**^

Group (T1)^a^	Households visited	People examined	New cases detected in T2 (%)^*^	Paucibacillary	Multibacillary
“Positive house”	113	483	84 (17.4 %)	27	57
“Negative house”	18	95	7 (7.4 %)	2	5
Total	131	578	91 (15.7 %)	29	62

* *p* < 0.05 - Fisher’s exact test

^a^“Positive house” = household with at least 1 seropositive dweller. “Negative house” = household with only seronegative dwellers

**Fig. 2:**
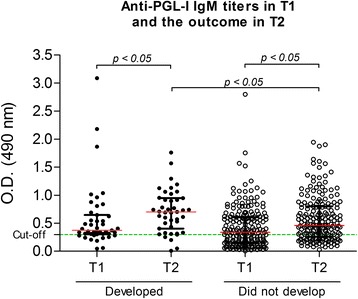
Anti-PGL-I IgM titres in the first (T1) and in the second (T2) evaluation. All HHC and SC that were evaluated twice (T1 and T2) are included in this analysis. Of 254 people examined, 43 (16.9 %) developed overt disease, and 211 did not by the 2-year follow-up. The most important increase in the IgM titres was observed in the group that developed the disease

Moreover, the group that did not develop leprosy during this period of time also demonstrated a significant increase in their average antibody titres (T1—median OD = 0.336, IQR = 0.461; T2—median OD = 0.460; IQR = 0.543; *p <* 0.05 by the Mann–Whitney *U* test). However, the most significant increase in the IgM titres was observed in the group that developed disease (T1—median OD = 0.371, IQR = 0.359; T2—median OD = 0.702, IQR = 0.562; *p <* 0.05 by the Mann–Whitney *U* test) (Fig. 2). Despite this result, 18/148 (12.1 %) of those who were seropositive at T1 became seronegative after 2 years, and 60/106 (56.6 %) of those who began as seronegative became seropositive, including seven that were diagnosed with leprosy.

### Seropositive versus seronegative houses

At T2, in addition to the 254 people evaluated twice, we also examined an additional 324 subjects that were not examined in T1, including both HHC of leprosy patients and HHC of seropositive or seronegative students. We operationally classified households with at least one seropositive dweller as “positive houses” and those with only seronegative dwellers as “negative houses”. An analysis of these additional subjects revealed an additional 48 (14.8 %) new cases for a total of 91. There was a significant difference (*p <* 0.05 by Fisher’s exact test) in the incidence of new cases among people from “positive houses” compared to those in “negative houses” (Table 3). The odds of finding a new leprosy case in “positive houses” was 2.6 times higher than in negative houses (95%CI = 1.18–5.91; *p <* 0.05), indicating that in a follow-up of 10 people living in “positive houses”, the probability of detecting at least one new case in a period of 2 years is 0.85 (or 85 %).

### Survey of students in high risk areas

We also evaluated an additional 134 students, aged 6–14 years (mean = 10.4) from two public elementary schools located in high-risk areas of Castanhal. Eleven (8.2 %) new leprosy cases were detected based on clinical signs and symptoms for disease. Four were classified as PB leprosy and seven as MB (4 BT and 3 BB). No physical disability was observed among these 11 cases; 4 (36.3 %) reported previous contact with at least one leprosy case (household or close contacts) ranging from 3 to 5 years long. Three individuals (27.2 %) did not show a BCG scar. The most frequent skin lesion was hypopigmented macules with loss of sensation.

A very high seroprevalence of anti-PGL-I IgM (104/134; 77.6 %) was observed in this sample of students (median OD value of seropositive SC was 0.564; IQR = 0.296), but 5 of 11 new cases (45.4 %) tested negative (1 Indeterminate, 1 primary neural, 2 BT and 1 BB). There was no significant difference (*p >* 0.2 - Mann–Whitney *U* test) between the median OD value of new cases (0.436; IQR = 0.287) and the median of healthy students (0.488; IQR = 0.337). We went to the residences of those SC who were newly diagnosed with leprosy and examined 42 of their HHC, and another 7 (16.6 %) new cases were identified with leprosy. Twenty-three of these HHC (54.7 %) also tested positive to anti-PGL-I (median OD value 0.657).

**Fig. 3:**
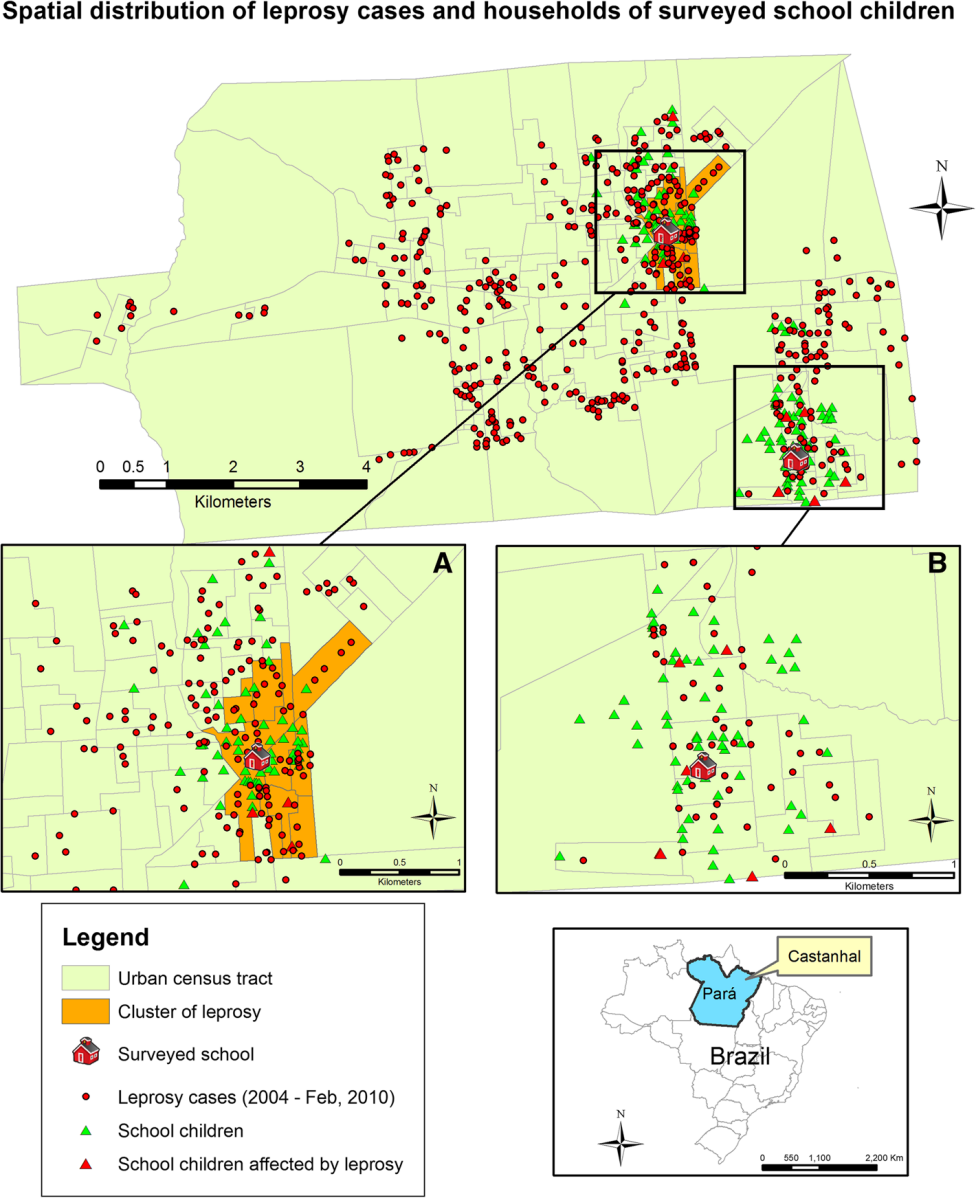
Spatial distribution of leprosy cases and households of surveyed school children in the urban area. We mapped 499 (87 %) of the total reported cases in the urban area of Castanhal, as detected from 2004 to February 2010 (SINAN database) and the residences of 134 examined school children. **a** The most likely cluster of leprosy cases determined by Kulldorff’s spatial scan statistics (orange area; *p* < 0.01); (**b**) A hyperendemic area in the periphery of the city (annual raw new case detection rate ≥40/100,000 people per census tract)

The spatial distribution of all leprosy cases reported in the SINAN database from 2004 to February 2010 was associated with the residence locations of the 134 evaluated SC (Fig. 3). We observed that 22 (16.4 %) were residing within 50 m of at least one leprosy case, 83 (62 %) within 100 m and 121 (90.3 %) within 200 m from a known case. All 11 new SC cases were living within 200 m of at least one case, 6 (54.5 %) of them within 100 m and 1 (9.1 %) within 50 m. There was a significant difference (*p <* 0.05 by Fisher’s exact test) between the proportion of new cases detected at the schools that were selected based on the spatial distribution of the reported cases (11 new cases of 134 SC; 8.2 %) and our previous findings [3] in randomly selected schools (63 new cases of 1592 SC; 3.9 %).

### Overall clinical and epidemiological outcomes

Of the 754 people included in this study, we detected a total of 109 (14.4 %) new cases; 40 (36.7 %) of these were children <15 years old. Of the 109 new cases, 95 (87.2 %) had DG 0 and 14 (12.8 %) had DG 1; 64 (58.7 %) were females; 91 (83.4 %) had at least one BCG scar. Sixty (55 %) were living in crowded houses (more than 2 dwellers per bedroom); the average number of people per household was 5.4, but in 9 houses (9.8 %), there were 10 or more dwellers. Among these people, 17 individuals (15.6 %) expected to move to another place in the near future, reflecting the highly mobile nature of people in this region. Sixteen (14.7 %) reported starvation at least one time in their lives, as defined by a full day without meals, because of the absence of resources to buy food; 55 (50.4 %) had a family income of equal to or lower than the Brazilian minimum monthly wage (roughly 250 US dollars) and 77 (70.6 %) received some type of financial assistance from the federal government, most often the family or school allowance (Brazilian official income transfer programs).

## Discussion

Performing targeted screening in selected schools located in a predefined cluster of leprosy cases or in a hyperendemic urban census tract of the city resulted in a two-fold higher detection rate compared to our previous findings in randomly selected schools [3]. All new cases detected among SC were from households in close proximity to reported cases. This spatial correlation can also help to understand the extremely high prevalence of subclinical infection observed in this sample of students because neighbours and extra-domiciliary contacts are associated with increased risk of leprosy as well [26, 27]. Simple serological assays used to detect anti-PGL-I IgM demonstrated their utility as an indicator for the high rate of infection in hyperendemic cities, and positive titres to PGL-I have been shown to be a biomarker of infection at the individual level, as well as a landmark of households with an increased risk of leprosy. Fine-scale spatial epidemiology and serology data should be collected to increase the detection rate in hyperendemic regions of the globe.

The strength of the antibody titre has a good correlation with the bacterial load [28, 29], and patient responses against PGL-I and other protein antigens, such as LID-1, have been demonstrated to predict the onset of leprosy in the armadillo model and in prospective longitudinal clinical settings [30–34]. Our analysis indicates a very high probability (>90 %) that at least 1 of 10 seropositive people will progress to overt disease within a period of 2 years and that antibody titres will significantly increase in most of those who will eventually develop the disease before they are diagnosed. However, seronegative HHC should not be neglected, especially in hyperendemic areas because anti-PGL-I serology tests have poor sensitivity (approximately 50 %) even to detect those with established PB leprosy [35].

Moreover, in this study, we showed that 2 years was a sufficient length of time for some seronegative individuals to become seropositive and develop clinical manifestations of leprosy. We observed a slight but statistically significant increase in the average antibody titres among those people who did not develop the disease during this follow-up. There is evidence that treating the index case in a household results in lower titres to *M. leprae* antigens in HHC residing in the household over time [30], as MDT therapy would cause a rapid cessation of shedding of viable bacilli, thus eliminating further exposure of HHC to mycobacterial antigens. Once the pattern of exposure has been broken, one would expect that antibody titres would decline in many of the HHC following successful MDT.

In addition to identifying those specific individuals with the highest titres to PGL-I, thus establishing their higher risk to succumb to disease, serologic data also enabled us to identify those households most at risk of leprosy. The probability of new cases in “seropositive houses” is more than two-fold higher compared to “negative houses”. We calculated that there is an 85 % probability that at least 1 of 10 people in these “positive houses” will progress to overt disease in a period of 2 years. Similar findings were obtained by a prospective study conducted in Cebu (Philippines), where HHC in approximately 1 of 7 households of MB leprosy patients developed leprosy during a 7-year period of active surveillance [36]. Based on their results, those authors suggested treating antibody-positive high-risk household contacts, even with no clinical manifestations, with an MB leprosy treatment regimen to prevent transmission. However, based on our field experience, this control approach does not appear practical in hyperendemic settings such as Pará, Brazil, where there is an extremely high seroprevalence rate of anti-PGL-I. Some researchers have tried chemoprophylaxis as an alternative strategy to interrupt the transmission of *M. leprae* in highly endemic settings, and one study showed that single dose rifampicin therapy provided approximately 60 % protection against the disease during the first 2 years [37–39]. However, this type of solution is not widely recommended because there are reservations regarding how long the protective effect is, the development of new resistant strains, and its efficacy in such hyperendemic areas that have a high prevalence of undiagnosed cases.

We were only able to re-evaluate 33.8 % of the original subjects surveyed in T1, which is a major limitation of this study. Moreover, more females than males were included in our surveys because women are frequently in charge of domestic tasks and were at home when we visited, whereas men usually work outside the home. Considering that global epidemiological data historically has shown a higher incidence of leprosy among males (in some studies as high as a 2:1 ratio of males to females) [40], we likely missed some cases during this study and underestimated the size of the problem. We classified a household as a “negative house” based only on dwellers that we evaluated, but in some cases, we were not able to examine all residents, which could also be a source of bias by not detecting possible seropositive individuals in those “negative houses”.

There is strong evidence that HHC and social contacts (at school, workplace, religious temples, etc.) and neighbours of leprosy cases have an increased risk of leprosy [26, 27, 41, 42]. Consequently, it has been suggested that contact surveys should focus not only on HHC but should also be extended to entire neighbourhoods or villages to target a greater spectrum of social contact networks. However, in a regional scenario where less than 50 % of HHC of reported leprosy cases were examined in the last 10 years, mainly because of the low coverage and the inefficiency of the local public health system in the state of Pará, this goal remains a challenge. More resources are needed to evaluate all HHC of new leprosy cases and to extend contact tracing to a wider network of people at higher risk of leprosy in a sustainable manner.

Spatial targeting has been applied to control various infectious diseases, including leprosy [43–45]. Surveys of school children in high or hyperendemic areas for leprosy has long been advocated as an important strategy for early detection since 1947 [46], but it is not generally recommended by either national and/or regional control programmes despite some evidence of its efficacy [47–51]. In a recent national student survey, the 2014 Brazilian leprosy campaign concentrated its strategy on evaluating school children of public schools from highly endemic municipalities of the country. It targeted 4.7 million students using an evaluation scheme in which the parents of the children were in charge of discovering suspicious skin lesions.

As a result, 199,087 students were clinically examined by physicians at the basic health units, 354 of them (0.17 %) were newly diagnosed with leprosy and about 100 new cases were detected among their household contacts (official data of the Ministry of Health, April 2015). Despite this particularly alarming detection rate among children, indicating active foci of infection in their communities, this number may be even higher. Those referred children were examined by general doctors, which are not always familiar with leprosy and therefore may not be skilled to define a child leprosy case. A child diagnosis is often challenging even for experienced leprologists. Furthermore, poorer areas and families are associated with a higher amount of leprosy cases, and these families have more difficulty finding a suspicious spot on the children’s skin.

## Conclusions

Performing targeted screening involving school-based active clinical surveillance in high risk areas determined by spatial epidemiology, accompanied by regular follow-up of targeted HHC and families guided by serologic data, significantly increases the likelihood of early detection of new leprosy cases.

Based on our findings, we strongly believe that if large-scale school children surveys are performed in specific spatial clusters of leprosy in each hyperendemic municipality with well-trained personnel, the detection rate would be much higher. We advocate such an approach both for public health reasons and because it will be more cost-effective than what has been conducted in Brazil.

## Abbreviations

WHO: World Health Organization
PGL-I: Phenolic glycolipid-I
HHC: Household contacts
IgM: Immunoglobulin M
GIS: Geographic information systems
S: South
W: West
NE: Northeast
SC: School children
T1: First evaluation
T2: Second evaluation
TT: Tuberculoid-tuberculoid
BT: Borderline tuberculoid
BB: Borderline-borderline
BL: Borderline-lepromatous
LL: Lepromatous-lepromatous
PB: Paucibacillary
MB: Multibacillary
DG: Disability grading
ELISA: Enzyme-linked immunosorbent assay
OD: Optical density
SINAN: National Notifiable Diseases Information System
IBGE: Brazilian Institute of Geography and Statistics
GPS: Global positioning system
NNH: Number needed to harm
SD: Standard deviation
IQR: Interquartile range
95%CI: 95 % confidence interval
BCG: Bacillus Calmette–Guérin
LID-1: Leprosy Infectious Diseases Research Institute Diagnostic-1
MDT: Multidrug therapy
